# Editorial: Demonstrating quality control (QC) procedures in fMRI

**DOI:** 10.3389/fnins.2023.1205928

**Published:** 2023-05-31

**Authors:** Paul A. Taylor, Daniel R. Glen, Richard C. Reynolds, Arshitha Basavaraj, Dustin Moraczewski, Joset A. Etzel

**Affiliations:** ^1^Scientific and Statistical Computing Core, National Institute of Mental Health, National Institutes of Health, Bethesda, MD, United States; ^2^Data Science and Sharing Team, National Institute of Mental Health, National Institutes of Health, Bethesda, MD, United States; ^3^Psychological and Brain Sciences, Washington University in St. Louis, St. Louis, MO, United States

**Keywords:** fMRI, quality control, resting state, task-based, visualization

## Introduction

This Research Topic, “*Demonstrating quality control (QC) procedures in fMRI*^1^,” focused on promoting quality control descriptions and discussions within the FMRI community. We invited anyone in the field to participate and perform their QC protocol of choice on sets of task-based and resting state FMRI data, describing their steps and criteria in detail. Ten teams participated, utilizing processing and QC methods that are available from a wide variety of software packages. The resulting set of articles represents a didactic resource for the field moving forward, as a reference for teaching and describing QC procedures.

The examined data collection came from real, unaltered, and publicly available datasets from widely used distributions. Even if a repository is curated, one would likely still expect to see some QC issues arise—that is one of the fundamental reasons this Research Topic was organized, and the aim of this project is certainly not to derogate the collections themselves but simply to use “real world” datasets for demonstrating detailed QC. The assortment was selected explicitly to include a full gamut of “good” to “poor” quality datasets. In the end, among the QC issues found and reported by the Project contributors were: extreme subject motion, severe ghosting, upside-down EPIs, incomplete FOV coverage, low TSNR, severe EPI distortion and dropout, left-right flipping of datasets, mismatched subjects, systematic spatio-temporal EPI artifacts, incorrect slice-timing, task-correlated motion, invalid task performance and anomalous correlation patterns. These are all issues that can affect study results, and this highlights how *anyone* undertaking an FMRI project should include careful QC assessments as part of their workflow.

Here we first describe how the focal data collection was assembled. We then give an overview of the software utilized, and highlight commonalities across the contributions of the participating teams, as well as differences and unique aspects of each. Finally, we present recommendations based on the accumulated Project contributions for the neuroimaging community around QC considerations, which apply when using either public data or acquiring one's own.

## Methods

### Project instructions for participants

We briefly describe the Project instructions for participants (see https://osf.io/qaesm/ for more details). Participants were asked to perform their preferred QC steps on provided task-based and/or resting state FMRI data collections, and to describe their evaluation criteria in detail, including representative examples. Researchers could choose any desired processing steps for a whole brain study, with the final EPI data aligned to a specified MNI template (see below). “Whole brain” included subcortical structures but excluded the cerebellum, as many datasets do not fully cover the latter.

The participants could perform any QC steps they would normally use for such an analysis, using any software, visualization or processing. Each analyzed subject's dataset would be placed into one of the following categories: “include” (passing all QC criteria; high confidence to use in a study); “exclude” (fails one or more QC criteria; high confidence to remove); and “uncertain” (questionable for whether to include).

For the Project write-ups, the participants were asked to explicitly list all their evaluative criteria, and to denote quantitative and qualitative ones. Additionally, authors should:

*Describe each item listed in the QC criteria table(s) in sufficient detail for others to apply the same criteria. The criteria may also be structured as a protocol. Write the descriptions in a didactic manner, as if explaining each item to a new research assistant. Please detail quantities used*.

Finally, each Project should contain a presentation of a variety of interesting and representative QC examples across each of the categories.

### Dataset selection

To facilitate the QC discussions, we created a single, common collection from public repositories for participating researchers to analyze. Here we list the source datasets, as well as the approach for selecting them.

We chose to start with example investigations of commonly used data, namely human acquisitions at 3T with a single echo, which have long formed the bulk of FMRI studies. For the Project's initial distribution of data, the acquisition site and original subject IDs were anonymized, to reduce possible evaluation biases. Since FMRI analysis is often performed on groups of subjects, and some QC factors might be considered “relative” to the group, subject ID numbering was used to identify sets of subjects from a particular site. Separate sites were labeled with group numbers, and subject IDs were simply remapped with the first digit reflecting group membership: Group 0 = sub-001, sub-002, …; Group 1 = sub-101, sub-102, …; etc. (see the table in the Supplementary material for the full mapping). No properties of the datasets (data values, header information, etc.) were altered in this process. The datasets are publicly available from the “FMRI Open QC Project” webpage^2^ (Taylor et al., 2023), which also contains further details of the Project description.

For the resting state collections, we browsed available data repositories that had open use agreements, including ABIDE-1 and ABIDE-2 (Di Martino et al., 2014), AOMIC (Snoek et al., 2021), Functional Connectome Project (FCP; Biswal et al., 2010), MPI-LEMON (Babayan et al., 2019), SALD (Wei et al., 2018), and SLIM (Liu et al., 2017), as well as a large number of OpenNeuro (Markiewicz et al., 2021) collections. In total, over 230 separate resting state data collections were initially examined for this project.

The first selection stage was to find collections with the following properties:

- Having >12 subjects, each of whom has at least one EPI and one T1w volume in the same session directory.
- EPI: TR > 1.5 s, all voxel edges < 4.1 mm, number of volumes > 100, non-zero srow values in the NIFTI header.^3^
- T1w: all voxel edges < 2.1 mm, non-zero srow values in the NIFTI header.

This reduced the number of collections to 56.

Then, quick processing and brief visual investigation were performed. Data collections with systemic issues, such as overly tight FOV (cutting off the cerebellar cortex), very poor EPI tissue contrast, obvious ghosting in the EPIs, and odd coordinate systems (e.g., not approximately centered around the coordinate origin, suggesting possible DICOM conversion and header issues) were removed from further consideration. From the remaining sets, we selected collections with a variety of voxel sizes, run lengths and numbers of runs, and particularly those that appeared to contain both reasonable data and a variety of occasional (but not systemic) QC considerations. To finalize the Project data collection size, we aimed to balance the breadth of data properties to explore with the number of researchers likely to participate: having more sites/subjects would likely increase the former but decrease the latter.

Therefore, we settled on having seven resting state FMRI sites from various data repositories and formed “groups” of ~20 subjects each. Most of the Project groups were subsets of their original repository collections; the subsets generally had a range of subject motion and other underlying considerations. Some repositories originally contained explicit categorization of subjects as “control” and non-control, such as having TBI (traumatic brain injury) or psychiatric diagnosis; those designations did not influence data selection, and subjects were typically drawn from multiple categorizations, as most MRI studies contain such combinations. The final list of included resting state datasets (Groups 1–7) is provided in Table 1, with a brief description of properties by site/group.

**Table 1:** List of the sites from which project datasets were selected, along with brief descriptions of EPI properties.

**Brief descriptions of the resting state datasets used in the project**
**Group 1:** ABIDE-1, KKI (Barber et al., 2012; Nebel et al., 2014), *N* = 20 subjects used (of 55 total). *FMRI acquisition details:* Philips Achieva 3T scanner, EPI axial slice acquisition with fat saturation and SENSE (factor=3), flip angle = 75°, TE = 30 ms, TR = 2.5 s, voxel size = 2.67 × 2.67 × 3.0 mm, slice timing provided in JSON sidecar, PE direction = j-; subjects instructed to focus on a crosshair on black computer screen.
**Group 2:** ABIDE-1, Trinity (Delmonte et al., 2012), *N* = 20 subjects used (of 49 total). *FMRI acquisition details:* Philips Achieva 3T scanner, EPI axial slice acquisition with fat saturation and SENSE (factor=2), flip angle = 90°, TE = 28 ms, TR = 2.0 s, voxel size = 3.0 × 3.0 × 3.841 mm, slice timing provided in JSON sidecar, PE direction = j-, subjects instructed to close eyes during scan.
**Group 3:** ABIDE-2, KUL-3 (Bernaerts et al., 2016), *N* = 16 subjects used (of 28 total). *FMRI acquisition details:* Philips Achieva Ds 3T scanner, EPI axial slice acquisition with fat saturation and with SENSE (factor=2), flip angle = 90°, TE = 30 ms, TR = 2.5 s, voxel size = 1.562 × 1.562 × 3.1 mm, slice timing provided in JSON sidecar, PE direction = j-, subjects instructed to focus on a white fixation cross on black background.
**Group 4:** FCP, Baltimore (Pekar and Mostofsky, 2010), *N* = 23 subjects used (of 23 total). *FMRI acquisition details:* 3T scanner (unspecified type), TR = 2.5 s, voxel size = 2.667 × 2.667 × 3.0 mm, subjects instructed to keep eyes open and fixate (target unspecified) during scan.
**Group 5:** OpenNeuro, ds000220 (Roy et al., 2017), *N* = 20 subjects used (of 26 total). *FMRI acquisition details:* Philips Achieva and Siemens Trio 3T scanners, EPI axial slice acquisition with segmented k-space (no SENSE), flip angle = 90°, TE = 34 ms, TR = 2 s, voxel size = 1.85 × 1.85 × 4.0 mm, instructions to subjects undescribed.
**Group 6:** OpenNeuro, ds000243 (Petersen et al., 2018), *N* = 20 subjects used (of 120 total). *FMRI acquisition details:* Siemens Magnetom Trio 3T scanner, 12 channel head coil, flip angle = 90°, TE = 34 ms, TR = 2.5 s, voxel size = 4.0 × 4.0 × 4.0 mm, instructions to subjects undescribed.
**Group 7:** OpenNeuro, ds000245 (Yoneyama et al., 2018), *N* = 20 subjects used (of 45 total). *FMRI acquisition details:* Siemens Verio 3T scanner, 12 channel head coil, flip angle = 80°, TE = 30 ms, TR = 2.5 s, voxel size = 3.0 × 3.0 × 3.51 mm, slice timing provided in JSON sidecar, subjects instructed to close eyes during scan.
**Brief description of the task-based state datasets used in the Project**
**Group 0:** OpenNeuro, ds000030, “task-pamenc” (Poldrack et al., 2016; Bilder et al., 2018), *N* = 30 subjects used (of 272 total). *FMRI acquisition details:* Siemens TrioTim 3T scanner, EPI acquisition with segmented k-space and fat saturation (acceleration factor PE = 2), flip angle = 90°, TE = 30 ms, TR = 2 s, slice timing provided in JSON sidecar, PE direction = j-.

See the Supplementary material for a detailed subject list from each site. In some cases, properties varied across the site, which was noted within some QC evaluations, and properties shown here are those for the first subject in each group. Group 4′s details were not provided in original project downloads, because the dataset JSON sidecar files did not contain acquisition information. This description comes from the “Release Table (April 6, 2012)” spreadsheet from the FCP download website: https://www.nitrc.org/docman/?group_id=296. For Groups 5–7, voxel size was included only in the NIFTI dataset, not included in the JSON sidecar.

Similar considerations to the above were used for selecting task-based FMRI data. As an additional factor, there are a wide variety of possible task designs, with differing degrees of complexity for modeling and analysis. Quality control considerations of the paradigm timing, both in terms of setup and subject response, are important in much of FMRI research. For this Project we decided to use task FMRI data from a single site and paradigm, and we wanted to select a relatively straightforward design with a small number of stimulus classes, to simplify explication, processing and modeling considerations.

Thirty subjects from the following task-based dataset were selected. Table 1 provides a brief description of this “Group 0,” including FMRI acquisition properties contained within the JSON sidecar files in the Project download. The specific task was a paired memory encoding task (“pamenc”) with button-pushing responses (see Poldrack et al., 2016, for details). In addition to the originally distributed events TSV file, we also provided a simplified task file with only three columns: stimulus onset time, duration and a trial type label (“TASK,” “CONTROL”). Teams were free to use either set of timing information—or even to not use any—as part of their QC. Onset timing was essentially identical for all but two subjects (whose onsets were uniformly 2 s later), separated by 2.5–18.5 s (mean = 7.5 s). Response times, which could represent event duration, had per-subject means of 0.51–1.57 s (range = 0.0–2.43 s) for CONTROL events and 0.45–2.65 s (range = 0.0–4.0 s) for TASK events. Inter-stimulus interval times ranged from 1.3 to 17.3 s (mean = 6.4 s).

We note that de-identifying the task data to fully blind teams from the source dataset was challenging, because BIDS (Brain Imaging Data Structure specification) encodes the task label explicitly in the dataset filenames. For example, an EPI dataset is called sub-001_task-pamenc_bold.nii.gz, where “pamenc” is the label for the specific task; searching online for “fmri pamenc” leads to the original repository. Because we did not want to change any dataset properties besides the subject IDs (to avoid introducing any errors by mistake), we neither relabeled columns within the subject timing files nor changed the task label in the filenames. Therefore, in theory, participating teams could have investigated more background details about the task data; we are unaware if any did, but, in practice, essentially the same QC considerations would still apply.

In the end, the available Project data collection was comprised of seven groups of 139 total resting state FMRI subjects and 1 group of 30 task-based FMRI subjects. Each subject had one T1w anatomical reference and 1 or 2 EPI functional runs from a single session directory. These collections were intended to provide a basis for QC examples, with a full spectrum of data quality within each group and a diverse assortment of items to discuss across the subjects: having a mix of both reasonable and poor quality data would facilitate clearer depictions of QC procedures and contrasts. The collections were initially investigated for this purpose, using a quick inspection. However, during the course of the analysis for the Project itself, it became apparent with a more complete QC procedure that the EPI datasets for two groups actually did contain systemic artifacts (see Reynolds et al.). While this is certainly worth examining and understanding from a QC point of view, it had not been the intention to include such datasets within this project. This occurrence does primarily highlight two important points: (1) an in-depth quality control investigation is necessary on at least some subset of a data collection to truly understand its contents, whether using shared or acquired data; and (2) QC must be performed *from the start of data acquisition* (also using an in-depth examination), to avoid the propagation of systemic issues.

The repositories from which subjects were drawn contained a wide range of age spans: from 8–13 to 56–78 years. Neither age nor sex nor any other subject-specific information was included in the accompanying participants.tsv file, as part of anonymization. In “real” FMRI studies that use a standard template to define a common final space, it is generally considered preferable to match that template to the age of the subjects, such as the Haskins pediatric template (Molfese et al., 2021) for studies of children; and, increasingly, templates and atlases exist for a wider variety of geographical locales, such as Korean (Lee et al., 2005), Chinese (Tang et al., 2010), and Indian (Holla et al., 2020) populations, which may also provide a better reference. However, since the present project was focused on subject-level QC considerations and not on a group-level report, researchers were asked to use just a single reference template for simplicity and uniformity: the widely used MNI-2009c ICBM152 T1w, non-linear asymmetric volume (Fonov et al., 2011). Any particularly notable mismatches to the template dataset would be deserving items for QC commentary by the participating teams.

### BIDS packaging

The selected datasets were then merged into BIDS-valid resting state and task-based collections. We used multiple versions of the BIDS validator (1.2.5 and 1.9.9) to ensure BIDS compliance. As noted above, we did not alter the data or metadata supplied from the source dataset. Since each of the datasets was already available publicly in a BIDS structure, we only needed to rename the directories and files according to our site-based enumeration (see Supplementary material).

We first merged the seven resting state groups into a single data collection, and then deposited the appropriate top-level text files (dataset_description.json, participants.^*^, etc.) into each of the resting state and task-based collections. For resting state Group 4, we noticed that the JSON sidecar for the functional image in the source dataset was provided at the dataset level instead of at the participant level. To maintain consistency with the other groups, we copied this sidecar to the latter and renamed the file accordingly. We also note that for resting state Groups 4 and 5, JSON sidecars for the T1w images had not been supplied in the source dataset. Since metadata fields contained in these sidecars are often contingent on conversion software version, we opted to preserve the absence of this metadata.

We found no validation errors in the resting state collection and noted five warnings: (1) some images were not supplied with slice timing info; (2) not all subjects contained the same number of EPI files (e.g., some subjects in Group 6 had two functional runs, while the rest of that group and all other groups only contained one per subject); (3) not all subjects/sessions/runs had the same scanning parameters, sometimes even within a single group/site; (4) NIFTI header fields for unit dimensions were missing in the anatomical volumes for some subjects (xyzt_units was 0 for most of Group 1 and all of Group 2); and (5) two subjects (sub-506 and sub-507) had a mismatch between the number of items in the SliceTiming array and the *k* dimension of the corresponding NIFTI volume. For the task-based collection we found no validation errors and one warning: the tabular file contained custom columns not described in the data dictionary for the timing files. We avoided altering any of these warnings, as they existed in the original data, and left these as possible QC items for teams to discuss.

### Participating teams and software utilized

One goal of this Research Topic was to have as wide a representation of software tools and research labs as possible, in order to have a maximal breadth of QC descriptions. The Research Topic was advertised widely on general MRI analysis message boards, such as the INCF's Neurostars, and on email lists, such as the open “niQC” email group, which was created to foster discussions on neuroimaging quality control. It was advertised at major neuroimaging conferences and workshops, such as ISMRM and OHBM. Email notices were also sent to members of software development groups, to project consortia (e.g., ENIGMA) and to many FMRI labs across the field. In the end, there were 10 participating teams, from labs across three continents.

Across the contributions, there was a wide array of software used for each of the processing and QC phases. We list the processing and QC software packages used by each team in Table 2. We note that virtually all of the tools and implemented procedures exist in freely available (and mostly open source) software. As a result, this means that this set of Topic contributions assembles detailed QC descriptions across many widely used software packages that can immediately be used across the field for training, processing and research applications.

**Table 2:** Software used by each participating team for data processing and quality control.

**Team**	**Software for processing**	**Software for QC**
(A) Birn	AFNI, FSL, ANTs	AFNI
(B) Di and Biswal	SPM, Matlab	SPM, Matlab
(C) Etzel	fMRIPrep (with ANTs, AFNI, FreeSurfer, FSL, Nipype)	R (with knitr, RNifti and fields), AFNI
(D) Lepping et al.	AFNI	AFNI, REDCap
(E) Lu and Yan	DPABI, DPABISurf, DPARSF, fMRIPrep, FreeSurfer, ANTs, FSL, AFNI, SPM, PALM, Matlab, DARTEL	DPABISurf, DPARSF, fMRIPrep, Matlab
(F) Morfini et al.	CONN (with ART), SPM12, Matlab	CONN, SPM12, Matlab, FSLeyes
(G) Provins et al.	MRIQC (with ANTs, AFNI, FreeSurfer, FSL, Nipype, SynthStrip), fMRIPrep (with ANTs, AFNI, FreeSurfer, FSL, Nipype)	MRIQC (with ANTs, AFNI, FreeSurfer, FSL, Nipype, SynthStrip), fMRIPrep (with ANTs, AFNI, FreeSurfer, FSL, Nipype)
(H) Reynolds et al.	AFNI, FreeSurfer	AFNI
(I) Teves et al.	FreeSurfer, AFNI	AFNI
(J) Williams et al.	FSL, cinnqc (with FSL and pyfMRIqc)	pyfMRIqc

Citations for each are included here, in alphabetical order: AFNI (Cox, 1996), ANTs (Avants et al., 2012), ART (Ardekani and Bachman, 2009), cinnqc (https://github.com/bwilliams96/cinnqc), CONN (Whitfield-Gabrieli and Nieto-Castanon, 2012; Nieto-Castanon, 2020), DARTEL (Goto et al., 2013), DPABI (Yan et al., 2016), DPABISurf (Yan et al., 2021), DPARSF (Yan and Zang, 2010), fields (Nychka et al., 2017), fMRIPrep (Esteban et al., 2019), FreeSurfer (Fischl and Dale, 2000), FSL (Smith et al., 2004), FSLeyes (McCarthy, 2022), knitr (Xie, 2014), Matlab (www.mathworks.com), MRIQC (Esteban et al., 2017), Nipype (Gorgolewski et al., 2011), PALM (Winkler et al., 2014), pyfMRIqc (Williams and Lindner, 2020), REDCap (Harris et al., 2009, 2019), RNifti (Clayden et al., 2020), SPM (https://www.fil.ion.ucl.ac.uk/spm/; Ashburner, 2012), and SynthStrip (Hoopes et al., 2022).

## Results

### Common themes across teams

There were several common themes running across the participating teams' analyses.

**Figure 1:**
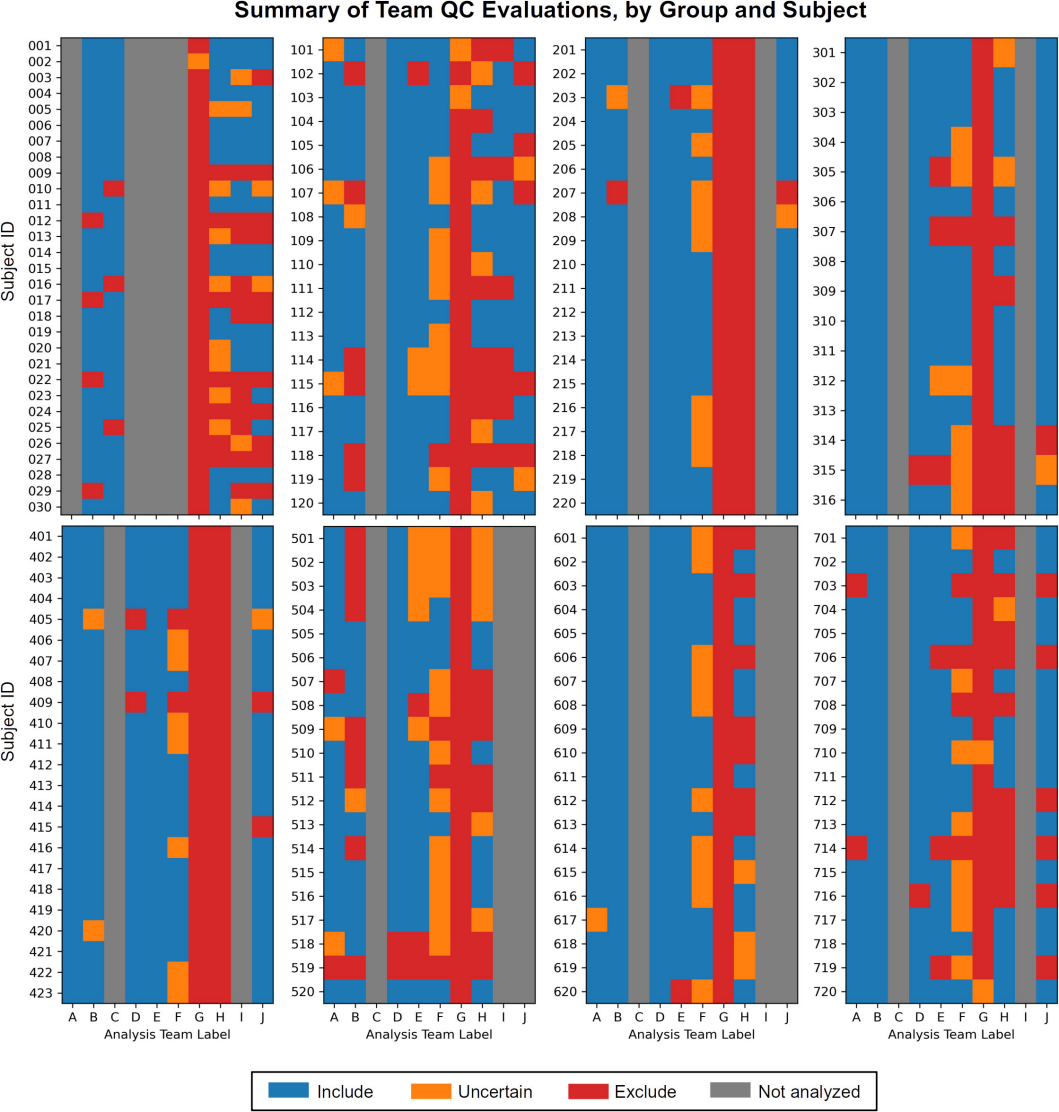
The set of QC evaluations for each subject (Group 0 = sub-001, sub-002, …; Group 1 = sub-101, sub-102, …; etc.), by each participating analysis team (see Table 2 for each column label, A–J). Group 0 contained task-based FMRI, and Groups 1–7 contained resting state FMRI. Groups within the data collection contained a range of data quality, from reasonable to poor. A large number of subjects were given evaluations of exclude or uncertain, showing the need for QC in FMRI studies. There is also variation among team evaluations, which was expected due to their different treatments of subject motion, signal coverage, and other focal features. This is discussed below in the Results.

**1) Each team found subjects to exclude based on one or more aspects of data quality**. As noted above, these collections all come from standard public data repositories. These repositories are great resources for the field for open data sharing, increasing multisite studies and having validation datasets, but there should generally *not* be the expectation that they are fully curated for data quality (and as noted in below in Theme 7, it may be impracticable to do so in a general way). Exclude-or-uncertain rating fractions varied across the teams, but many excluded 25% or more (Figure 1). In some cases, subtle but systematic artifacts were even found that led to recommending the complete exclusion of Groups 2 and 4 (see Reynolds et al.). These findings stress the importance of performing QC: *researchers should always check that data contents are appropriate for their study, whether acquiring collections themselves or downloading them*.

**2) Each team evaluated one or more subject's datasets as “uncertain.”** This is reasonable and expected, particularly when first investigating a data collection. This categorization would almost by definition be expected to be heterogeneous across researchers, given their different backgrounds, experience, opinions, expectations and intended use for the data. QC considerations and criteria will adapt over time, likely reducing the number of uncertain evaluations, but it is still a useful categorization to have in a QC procedure. It is essential to identify unknown or “surprising” features of a data collection or processing procedure. In a real study, this rating would likely be a temporary evaluation that leads to checking acquisition or other aspects more in-depth, perhaps even leading to a corrective measure or change in the acquisition. A subject given this rating may eventually be evaluated as either include or exclude.

**3) Nearly all QC protocols started by investigating the unprocessed data's consistency and “metadata” properties**. These included checking the number of EPI runs, voxel sizes, acquisition parameters, and other properties that are generally contained in the NIFTI headers and/or JSON sidecars; standard data collections are also likely to be accompanied by further subject descriptions (age, demographic, etc.). Even when acquiring one's own data, it is necessary to be sure that these underlying properties are consistent and meet expectations. Alterations in scanner settings, software version, DICOM field conversion and more can easily occur, and these can detrimentally alter dataset features, affecting final results or compatibility for inclusion within a study.

**4) Each team identified consistency, reliability or mismatch errors within subject datasets**. For example, all teams found two datasets that had upside-down EPI datasets, and some also identified left-right flip errors between a subject's EPI and anatomical volumes, which is a disturbingly common problem in FMRI (see Glen et al., 2020). Two teams even suspected that a subject's EPI and anatomical volumes came from different subjects, based on sulcal and gyral pattern mismatch. These kinds of fundamental data issues are difficult, if not impossible, to reliably correct after the fact. Some groups chose to address the EPI-anatomical consistency issues by assuming the anatomical dataset was correct, but while that may produce EPI-anatomical consistency, the presence of such header problems greatly reduces the reliability in absolute left-right identification. As was noted by multiple teams, fiducial markers are needed for clear identification (and some were identified in the visual inspections of some Project datasets here).

**5) Each QC protocol used qualitative criteria and visual inspection of datasets**. These included checking the raw data and inspecting derived images (e.g., TSNR or standard deviation maps) for suitability, as well as for artifacts. Visual checks were also used to evaluate the success of processing steps, such as alignment or statistical modeling. While these procedures cannot be performed automatically, they benefit greatly from systematization within a QC protocol, which software developers aim to facilitate. These qualitative checks carry the requirement for researchers to learn how to distinguish reasonable and problematic data, as well as to accurately communicate their methodology.

**6) Most, but not all, protocols included quantitative/automatic checks**. The teams employed a variety of quantities based on subject motion, TSNR and other measures. These tests are useful and find some of the most common kinds of expected problems. It was perhaps surprising that not every protocol included quantitative checks (while all *did* include qualitative ones, noted in Theme 5). This may reflect that visualization is still key to evaluating several data features and processing steps, and quantitative criteria typically originate as useful extensions of such understanding. It is likely that more developments for automating certain checks will be made over time, but this process also typically is rooted in visual inspection during the “training phase” of determining meaningful quantities and reasonable thresholds.

**7) QC parameters were closely tied to a specific analysis and research goal**. Nearly every group made the point that some subject data and data collections may be appropriate for one particular analysis but not another. As a consequence, it is likely not possible to simply adopt existing QC ratings on a given data collection from a separate study when using that data for a new project. While prior QC evaluations may inform those of a new analysis, the burden is always on the researcher to be sure of the contents of the data for their current application. There is no “one size fits all” set of criteria, as there is no single method for designing a study (sample size, number of groups, task paradigm, etc.), acquiring data (different field strengths, echo number, etc.), performing analysis (ROI- vs. voxel-based; surface vs. volumetric; etc.) and so on.

**8) Non-EPI items can affect FMRI analysis, too**. While the vast majority of QC evaluations focus on the EPI volume and its spatiotemporal properties, checks on the accompanying data can also affect the usability of the dataset as a whole. For example, some cases of notable anatomical variability were cited by most teams, such as having extremely large ventricles (and its limiting effect on the accuracy of template registration), as well as other anatomical anomalies. In other data collections one might find alterations to structure due to tumors, surgery or hemorrhages, which might necessitate removing a subject from the analysis or at least constrain the analysis options. Similarly, evaluating the stimulus timing in its own right was shown to be useful (Etzel; Reynolds et al.). For more complicated study designs, one might also analyze accompanying data such as physiological time series (such as cardiac and respiratory rate), etc. All the input data used for analysis needs to be reviewed.

**9) Each team made their processing and QC pipelines publicly available**. This kind of open processing (e.g., using GitHub, OSF or another accessible webpage) is becoming more common within the field, but it is important for this practice to keep growing. Given the didactic nature of this Research Topic, we hope that having these methods directly available will encourage the implementation for more detailed QC protocols and reporting.

### Individual elements and focuses among teams

Each of the submissions also introduced their own unique perspective and tools for quality control. We briefly list some examples here.

Birn analyzed the seven resting state groups. This paper explored the effects of using different motion thresholds, as well as the inclusion/exclusion of low-frequency fluctuation bandpassing, during processing. In particular, it investigated some trade-offs of trying to remove artifactual features with reducing the degrees of freedom in each subject's data, using network based dissimilarity matrices of QC-FC (Ciric et al., 2017; see below) that can be used for quality control evaluation.

Di and Biswal analyzed 1 task group (using stimulus timing) and the seven resting state groups. The authors included tissue-based segmentation estimates within their visual checks of anatomical-to-template volumetric registration. Tissue-masks were also used within a set of time-series checks of subject motion-related artifacts, where principal components of white matter and cerebrospinal fluid masks were examined for similarity with motion regressors and global mean signals.

Etzel analyzed 1 task group (using stimulus timing). This work focused on the task-based FMRI data. Among other QC steps, it included checks for participant behavior and responsiveness, such as by basing some criteria on patterns of button-pushing. Being confident that subjects had followed the task assignment to a reasonable degree is indeed paramount in neuroimaging, whether for explicit task-based paradigms or for naturalistic and resting state ones (with eye-tracking, alertness monitoring, etc.).

Lepping et al. analyzed the seven resting state groups. While all teams had an explicit list of QC criteria, this team created a REDCap checklist form to itemize and store the dataset assessments. They emphasized how this system facilitated the training and replicability aspects of the QC, which are vital aspects in any evaluation procedure. This also provided a convenient mechanism for sharing QC results.

Lu and Yan analyzed the seven resting state groups. This team included surface-based processing and criteria as part of their QC procedure, even though the analysis itself was explicitly volumetric. This allowed the evaluation to contain an interesting intersection of anatomy- and function-based assessment. They also briefly explored the differences of group-level tests with and without incorporating their excluded subjects.

Morfini et al. analyzed the seven resting state groups. Among other QC criteria, this team used multiple “QC-FC” analyses (Ciric et al., 2017) to evaluate the data at the group-level, an approach which incorporates both the quality of underlying data itself and the denoising/processing steps utilized on it. For example, one QC-FC measure involved calculating correlation matrices from 1,000 random voxels across a gray matter template in standard space.

Provins et al. analyzed one task group (not using stimulus timing) and the seven resting state groups. This work included exclusively qualitative assessments of quality, including signal leakage from eye movements, carpet plots and ICA-based components. One particular point of emphasis was on the importance of examining “background” features within the field of view (FOV), as patterns there can reveal several kinds of artifacts, such as aliasing ghosts, subject motion spikes, or scanner issues.

Reynolds et al. analyzed one task group (using stimulus timing) and the seven resting state groups. This QC procedure was organized into 4 or 5 separate stages for the resting state and task FMRI data, respectively, including GUI-based checks with InstaCorr (interactive “instant correlation;” Song et al., 2017) to follow up on observed spatio-temporal features as necessary. The authors also explicitly placed QC within the larger context of understanding the contents of the dataset and having confidence in what goes into the final analysis, rather than viewing it simply as a subject selection/rejection filter.

Teves et al. analyzed one task group (using stimulus timing) and one resting state group. This team organized their work as a QC assessment guide for both new and experienced researchers, and they emphasized the importance of interactive training and discussions with new researchers. They also used the comparison of EPI-anatomical alignment cost function values as a measure to trigger a visual check for potentially mismatched datasets.

Williams et al. analyzed one task group (not using stimulus timing) and five resting state groups. In particular, this work focused on the issue of inter-rater variability and reliability. Even within a single lab performing QC, there can be different assessments of datasets: qualitative evaluations can vary, as well as the choice of specific quantitative thresholds. This issue is also critical for describing QC procedures as accurately as possible to others when reporting results.

### General differences in team perspectives

Overall, there were some general differences in teams' approaches and scopes, which influenced QC discussions and selections. These did not reflect decisions that would necessarily be described as either right or wrong, but rather different choices made by teams that would contribute to variability of dataset evaluations (see Figure 1).

Firstly, the range of QC items necessarily depended on what processing steps were implemented, and the latter choice can vary widely across the field of FMRI analysis. There is no generally defined set of processing steps to apply when performing QC of an FMRI dataset. For example, some groups included subject-level (or “first level”) regression modeling within their processing, while others did not.^4^

Secondly, for the task FMRI dataset, some teams chose to ignore the timing files in their QC processing, while others included the stimulus information. Some even analyzed and interpreted the performance information in detail on its own within the list of exclusion criteria (e.g., Etzel). These again reflect different choices and degrees for understanding the presented data, and will necessarily contribute to variability in subject selections. For more complicated task designs (which certainly exist within the field), one would expect the QC approaches to have further variability, and to be closely tied to the analyses at hand, such as which stimulus contrasts are particularly central to the analysis.

Thirdly, the issue of subject motion was viewed and treated differently. Some teams used estimated motion-based parameters (e.g., Enorm or FD) for censoring (or “scrubbing”) time points to remove potentially contaminated volumes, and then to include the number of censored volumes within subject exclusion criteria. Other teams adopted processing approaches to mitigate effects of subject motion in other ways (within minimal or no censoring), with the stated aim of avoiding potential biases, arbitrary thresholds and loss of subject data. These philosophical choices will result in very different criteria for QC evaluations, given that typical data collections contain a range of subject motion profiles.

Fourthly, there were also different interpretations of how much signal dropout and distortion within a volume was acceptable before excluding a subject. For example, one team excluded 166 out of 169 subjects (and listed the remaining 3 as uncertain) from the evaluations of these features (Provins et al.). In a real study, this consideration might take the form of listing brain regions of particular interest and verifying the signal quality there specifically.

Additionally, beyond the fact that researchers make their own choices when determining what data are satisfactory to include in their research, the Project guidelines omitted details such as research goals, which might imply anatomical regions of particular interest. Similarly, subject group types were omitted, which might identify subjects for whom elevated levels of typical motion, or anatomical anomalies, would be expected. Researchers also made independent choices on how to treat within-group inhomogeneity of acquisition, such as whether subjects were required to be scanned on the exact same grids or to have the same number of EPI runs. As such, for this Project variance in QC perspectives was expected.

## Discussion

The immediate goals of this project were:

1. **To promote the broader adoption of quality control practices in the FMRI field**. There are many QC tools and protocols available in publicly available software (e.g., those in AFNI, CONN, DPARSF, fMRIprep, MRIQC, pyfMRIqc, and SPM were all used here), perhaps more than people have typically realized, and this set of Research Topic articles provides a didactic collection of them for researchers and trainees to use.
2. **To facilitate the inclusion of more details in QC protocol descriptions**. Each Project contribution contained an explicit list of QC criteria, along with demonstrations of most features. We hope these help start to systematize QC reporting within the field.
3. **To develop the view of QC as more than “just” vetting datasets, but rather as more deeply understanding the contents of the collection and analysis as a whole**. This should allow for greater confidence in final results, and hopefully improve reproducibility and reliability across the field.
4. **To share QC criteria across researchers who are performing analysis and developers creating tools, thereby improving the set of available QC tools in the process**. We would expect increasing clarity and potentially homogeneity of QC methods as a result of this work.

One longer term goal is to motivate the development of new QC methods, techniques and criteria. As noted in several Project papers, MRI acquisitions and analyses are complex and always changing, so evaluation criteria should continue to adapt. For instance, new images may summarize a feature in a clearer way, or more quantitative methods could be developed to streamline QC procedures. It is our experience as methods researchers and software developers that these kinds of advances are often rooted in visualization and understanding: *quantitative checks are essentially extensions of qualitative ones, in which understanding is rooted*. The present project collected a large number of datasets with varied properties, so that many people could view and comment on them in detail—we hope this provides a useful incubator for further QC development, which can be expanded across more data collections and researchers.

Another long term goal of this project is to facilitate the development of a common language and clear description of QC items. Several teams noted that there is not currently any general commonality in criteria or descriptions in the field, and that developing one would improve the ability to use, understand and communicate QC in work. For example, even referring to an apparently straightforward mathematical measure like TSNR (temporal signal-to-noise ratio) can lead to confusion, since there can be multiple reasonable definitions. Therefore, analysts should specify which definition they are using (as well as ensure that they are using a reasonable one), not only a numerical threshold.

### QC recommendations for researchers

The following are recommendations for implementing quality control in FMRI studies, drawing from the accumulated Project contributions, guidance and suggestions.

**1) Check new acquisitions immediately—delays can lead to wasted data**. Performing in-depth evaluations is crucial with the first few subjects in a protocol, to avoid systematic errors from the start. Maintaining checks remains important as scanner settings can easily be changed by accident or through an upgrade, etc., and this can flag alterations in data quality or properties. Acquiring good data is always better than trying to fix problematic data retrospectively.

**2) Conduct detailed QC checks whenever using a new data collection or starting a new project**. Most public repositories explicitly note that curation should not be assumed, and prior checks may have focused on different purposes, regions of interest or type of analysis. QC also integrates directly with verifying processing steps, and different analyses may have different properties and requirements.

**3) Treat QC as understanding data, not just “removing bad data.”** FMRI datasets are complicated, and many small details can affect downstream results. Treating QC as purely the elimination of bad data can lead to selection bias and to missing systematic issues—often checking *why* some datasets get removed provides useful insight into the entire collection. Understanding the full properties (and realistic limitations) of data will generally lead to better interpretation of it. Researchers should be confident in their data and its contents, and in-depth QC is the only way to achieve this.

**4) Apply both qualitative and quantitative checks**.

Visual verifications remain fundamental in data analysis, as shown by the participating teams here. These can be usefully systematized for maximal efficiency and utility, and these inform and complement automatic checks of derived quantities. This combination also typically helps with the development of new QC measures.

**5) Clearly define and describe all QC steps and measures**. This is necessary to maintain consistency of the QC within a lab or group setting, as evaluations of features can change over time or differ among people. All quantities should be clearly defined, since there may be multiple derivations; thresholds are not useful if their associated quantities are not clearly described. Having clear checklists facilitates implementing the QC, as well as reporting it in papers and presentations.

**6) Coordinate QC evaluations with the paradigm and aims of the current study**. In practice, it is difficult to make one QC evaluation apply to all possible purposes, due to the variability of study design, regions of interest, etc. Viewing previous QC evaluations might be useful, but those could be missing important characteristics for the present work or be overly harsh/lenient. Include explicit QC discussions in the planning stages of each study design.

**7) Ensure (at least some) in-depth QC, even for large studies**. The typical amount of time, expense and per-researcher effort of acquiring any subject is large (e.g., planning, piloting, grant writing, training, acquiring, and analyzing). As many QC steps are already integrated into analysis software, the relative effort of checking data and processing quality is actually quite small compared to that of the other stages of acquisition and analysis—*QC should not be skipped simply because it comes near the end of processing*. Big data can still be corrupted by systematic issues in acquisition and analysis. Even when applying automatic checks across all subjects, in-depth QC (including visualization and qualitative checks) should still be applied to at least meaningful subsamples across scanners and systematically across time, to avoid wasted data and resources as well as artifactual results.

**8) Share QC advice and recommendations**. Stating what QC steps are most useful for identifying certain features or for validating data for certain analyses benefits everyone in the neuroimaging community. Similarly, adding new tests and features helps other researchers and software developers directly.

**9) Make QC scripts public where possible**. While textual descriptions of methods in papers are useful and provide explanatory context, there are many influential details for both processing and QC that exist only at the level of code. Researchers presenting findings in posters, talks and other presentations would also be encouraged to provide links to their processing and QC scripts. Having the code available provides a valuable resource for the field, and hopefully this will help promote the wider adoption of QC integration into FMRI processing.

**10) Make QC evaluations public where possible**. Many of the QC protocols and software tools implemented in Project contributions produced reports that can be shared and/or archived. These include PDFs, HTML pages, RedCap reports, and JSON files. These could be included in NeuroVault uploads, for instance, as well as linked to papers, presentations and data repositories. Additionally, provide QC feedback to public repository hosting sites and/or to the researchers who acquired the original data: just like software packages, data collections have version numbers because fixes and updates can be required; QC feedback can benefit the neuroimaging community.

**11) Stay up to date with QC developments**. QC measures and methods will change over time. New acquisition and analysis approaches will lead to new artifacts and other considerations to evaluate; new ideas and software developments provide new checks and solutions.

## Conclusions

This Project demonstrates that there are many tools and procedures currently available for performing quality control in FMRI. It also presents a healthy warning that much can go wrong with the complex data acquisitions and analyses that go into FMRI, and QC should be included in all studies, whether researchers are using public datasets or acquiring their own scans. With careful preparation and quality control investigations, researchers can be more confident that their results are based on reasonable data and the intended processing. In short, we urge researchers to choose a quality control method that is thorough and understandable, and to keep looking at the data.

## Author contributions

PT, DG, RR, and JE conceived of this project, organized it, and contributed to writing this article. AB and DM helped organize, package and distribute the datasets, and further contributed in writing this article. All authors contributed to the article and approved the submitted version.

## Footnotes

^1^ https://www.frontiersin.org/research-topics/33922/demonstrating-quality-control-qc-procedures-in-fmri

^2^doi: 10.17605/OSF.IO/QAESM: https://osf.io/qaesm/.

^3^That is, have a defined voxel grid, where the affine sform matrix in the NIFTI header is nonzero.

^4^In some cases, this may reflect a differentiation of “processing” vs “preprocessing,” in which the former includes subject-level regression while the latter does not. However, these terms are not used consistently across the field. Within the Project description, “processing” was consistently used.

## References

[B1] Ardekani B. A. Bachman A. H. (2009). Model-based automatic detection of the anterior and posterior commissures on MRI scans. Neuroimage 46, 677–682. 10.1016/j.neuroimage.2009.02.030 19264138 PMC2674131

[B2] Ashburner J. (2012). SPM: A history. Neuroimage 62, 791–800. 10.1016/j.neuroimage.2011.10.025 22023741 PMC3480642

[B3] Avants B. B. Epstein C. L. Grossman M. Gee J. C. (2012). Symmetric diffeomorphic image registration with cross-correlation: Evaluating automated labeling of elderly and neurodegenerative brain. Med. Image Anal. 12, 26–41. 10.1016/j.media.2007.06.004 17659998 PMC2276735

[B4] Babayan A. Erbey M. Kumral D. Reinelt J. D. Reiter A. M. F. Röbbig J.. (2019). A mind-brain-body dataset of MRI, EEG, cognition, emotion, and peripheral physiology in young and old adults. Sci. Data 6, 180308. 10.1038/sdata.2018.308 30747911 PMC6371893

[B5] Barber A. D. Srinivasan P. Joel S. E. Caffo B. S. Pekar J. J. Mostofsky S. H.. (2012). Motor “dexterity”?: Evidence that left hemisphere lateralization of motor circuit connectivity is associated with better motor performance in children. Cereb. Cortex 22, 51–59. 10.1093/cercor/bhr062 21613469 PMC3236793

[B6] Bernaerts S. Prinsen J. Dillen C. Berra E. Brams S. Wenderoth N.. (2016). Oxytocin-Based Pharmacotherapy for Autism Spectrum Disorders: Investigating the Immediate and Long-Term Effects From a Neural and Behavioral Perspective. Baltimore, MD: International Meeting for Autism Research (IMFAR).

[B7] Bilder R. Poldrack R. Cannon T. London E. Freimer N. Congdon E.. (2018). UCLA Consortium for Neuropsychiatric Phenomics LA5c Study. OpenNeuro. [Dataset] 10.18112/openneuro.ds000030.v1.0.0

[B8] Biswal B. B. Mennes M. Zuo X. N. Gohel S. Kelly C. Smith S. M.. (2010). Toward discovery science of human brain function. Proc. Natl. Acad. Sci. U. S. A. 107, 4734–4739. 10.1073/pnas.0911855107 20176931 PMC2842060

[B9] Ciric R. Wolf D. H. Power J. D. Roalf D. R. Baum G. L. Ruparel K.. (2017). Benchmarking of participant-level confound regression strategies for the control of motion artifact in studies of functional connectivity. Neuroimage 154, 174–187. 10.1016/j.neuroimage.2017.03.020 28302591 PMC5483393

[B10] Clayden J. Cox R. W. Jenkinson M. (2020). RNifti: Fast R and C++ *Access to NifTI Images*. Available online at: https://CRAN.R-project.org/package=RNifti (accessed May 24, 2023).

[B11] Cox R. W. (1996). AFNI: Software for analysis and visualization of functional magnetic resonance neuroimages. Comput. Biomed. Res. 29, 162–173. 10.1006/cbmr.1996.0014 8812068

[B12] Delmonte S. Balsters J. H. Gallagher L. (2012). Social and Monetary Reward Processing in Autism Spectrum Disorders (ASD): Interaction Effects in the Striatum. Toronto, ON: International Meeting for Autism Research (IMFAR). 10.1186/2040-2392-3-7 PMC3499449 23014171

[B13] Di Martino A. Yan C. G. Li Q. Denio E. Castellanos F. X. Alaerts K.. (2014). The autism brain imaging data exchange: Towards a large-scale evaluation of the intrinsic brain architecture in autism. Mol. Psychiatry. 6, 659–667. 10.1038/mp.2013.78 23774715 PMC4162310

[B14] Esteban O. Birman D. Schaer M. Koyejo O. O. Poldrack R. A. Gorgolewski K. J.. (2017). MRIQC: Advancing the automatic prediction of image quality in MRI from unseen sites. PLoS ONE 12, e0184661. 10.1371/journal.pone.0184661 28945803 PMC5612458

[B15] Esteban O. Markiewicz C. J. Blair R. W. Moodie C. A. Isik A. I. Erramuzpe A.. (2019). fMRIPrep: A robust preprocessing pipeline for functional MRI. Nat. Methods 16, 111–116. 10.1038/s41592-018-0235-4 30532080 PMC6319393

[B16] Fischl B. Dale A. M. (2000). Measuring the thickness of the human cerebral cortex from magnetic resonance images. Proc. Natl. Acad. Sci. U. S. A. 97, 11050–11055. 10.1073/pnas.200033797 10984517 PMC27146

[B17] Fonov V. Evans A. C. Botteron K. Almli C. R. McKinstry R. C. Collins D. L.. (2011). Unbiased average age-appropriate atlases for pediatric studies. Neuroimage 54, 313–327. 10.1016/j.neuroimage.2010.07.033 20656036 PMC2962759

[B18] Glen D. R. Taylor P. A. Buchsbaum B. R. Cox R. W. Reynolds R. C. (2020). Beware (surprisingly common) left-right flips in your MRI data: an efficient and robust method to check MRI dataset consistency using AFNI. Front. Neuroinformatics 14. 10.3389/fninf.2020.00018 32528270 PMC7263312

[B19] Gorgolewski K. Burns C. D. Madison C. Clark D. Halchenko Y. O. Waskom M. L.. (2011). Nipype: A flexible, lightweight and extensible neuroimaging data processing framework in python. Front. Neuroinform. 5, 13. 10.3389/fninf.2011.00013 21897815 PMC3159964

[B20] Goto M. Abe O. Aoki S. Hayashi N. Miyati T. Takao H.. (2013). Diffeomorphic Anatomical Registration Through Exponentiated Lie Algebra provides reduced effect of scanner for cortex volumetry with atlas-based method in healthy subjects. Neuroradiology 55, 869–875. 10.1007/s00234-013-1193-2 23619702

[B21] Harris P. A. Taylor R. Minor B. L. Elliott V. Fernandez M. O'Neal L.. (2019). The REDCap consortium: Building an international community of software platform partners. J. Biomed. Inform. 95, 103208. 10.1016/j.jbi.2019.103208 31078660 PMC7254481

[B22] Harris P. A. Taylor R. Thielke R. Payne J. Gonzalez N. Conde J. G.. (2009). Research electronic data capture (REDCap)–a metadata-driven methodology and workflow process for providing translational research informatics support. J. Biomed. Inform. 42, 377–381. 10.1016/j.jbi.2008.08.010 18929686 PMC2700030

[B23] Holla B. Taylor P. A. Glen D. R. Lee J. A. Vaidya N. Mehta U. M.. (2020). A series of five population-specific Indian brain templates and atlases spanning ages 6-60 years. Hum. Brain Mapp. 41, 5164–5175. 10.1002/hbm.25182 32845057 PMC7670651

[B24] Hoopes A. Mora J. S. Dalca A. V. Fischl B. Hoffmann M. (2022). SynthStrip: Skull-stripping for any brain image. Neuroimage 260, 119474. 10.1016/j.neuroimage.2022.119474 35842095 PMC9465771

[B25] Lee J. S. Lee D. S. Kim J. Kim Y. K. Kang E. Kang H.. (2005). Development of Korean standard brain templates. J. Korean Med. Sci. 20, 483–488. 10.3346/jkms.2005.20.3.483 15953874 PMC2782208

[B26] Liu W. Wei D. Chen Q. Yang W. Meng J. Wu G.. (2017). Longitudinal test-retest neuroimaging data from healthy young adults in southwest China. Sci. Data 4, 170017. 10.1038/sdata.2017.17 28195583 PMC5308199

[B27] Markiewicz C. J. Gorgolewski K. J. Feingold F. Blair R. Halchenko Y. O. Miller E.. (2021). The OpenNeuro resource for sharing of neuroscience data. Elife 10, e71774. 10.7554/eLife.71774.sa2 34658334 PMC8550750

[B28] McCarthy P. (2022). FSLeyes (1.5.0). Zenodo. 10.5281/zenodo.7038115

[B29] Molfese P. J. Glen D. Mesite L. Cox R. W. Hoeft F. Frost S. J.. (2021). The Haskins pediatric atlas: A magnetic-resonance-imaging-based pediatric template and atlas. Pediatr. Radiol. 51, 628–639. 10.1007/s00247-020-04875-y 33211184 PMC7981247

[B30] Nebel M. B. Joel S. E. Muschelli J. Barber A. D. Caffo B. S. Pekar J. J.. (2014). Disruption of functional organization within the primary motor cortex in children with autism. Hum. Brain Mapp. 35, 567–580. 10.1002/hbm.22188 23118015 PMC3864146

[B31] Nieto-Castanon A. (2020). Handbook of Functional Connectivity Magnetic Resonance Imaging Methods in CONN. Hilbert Press. 10.56441/hilbertpress.2207.6598

[B32] Nychka D. Furrer R. Paige J. Sain S. (2017). Fields: Tools for Spatial Data. Boulder, CO: University Corporation for Atmospheric Research.

[B33] Pekar J. J. Mostofsky S. H. (2010). FCP Classic Data Sharing Samples: Baltimore site Download. Available online at: http://www.nitrc.org/frs/downloadlink.php/1600 (accessed May 24, 2023).

[B34] Petersen S. Schlaggar B. Power J. (2018). Washington University 120. OpenNeuro. Available online at: https://openneuro.org/datasets/ds000243/versions/00001 (accessed May 24, 2023).

[B35] Poldrack R. A. Congdon E. Triplett W. Gorgolewski K. J. Karlsgodt K. H. Mumford J. A.. (2016). A phenome-wide examination of neural and cognitive function. Sci. Data 3, 160110. 10.1038/sdata.2016.110 27922632 PMC5139672

[B36] Roy A. Bernier R. A. Wang J. Benson M. French J. J. Jr. Good D. C.. (2017). The evolution of cost-efficiency in neural networks during recovery from traumatic brain injury. PLoS ONE 12, e0170541. 10.1371/journal.pone.0170541 28422992 PMC5396850

[B37] Smith S. M. Jenkinson M. Woolrich M. W. Beckmann C. F. Behrens T. E. Johansen-Berg H. M.. (2004). Advances in functional and structural MR image analysis and implementation as FSL. Neuroimage 23(Suppl.1), S208–S219. 10.1016/j.neuroimage.2004.07.051 15501092

[B38] Snoek L. van der Miesen M. M. Beemsterboer T. van der Leij A. Eigenhuis A. Steven Scholte H.. (2021). The Amsterdam Open MRI Collection, a set of multimodal MRI datasets for individual difference analyses. Sci. Data 8, 85. 10.1038/s41597-021-00870-6 33741990 PMC7979787

[B39] Song S. Bokkers R. P. H. Edwardson M. A. Brown T. Shah S. Cox R. W.. (2017). Temporal similarity perfusion mapping: A standardized and model-free method for detecting perfusion deficits in stroke. PLoS ONE 12, e0185552. 10.1371/journal.pone.0185552 28973000 PMC5626465

[B40] Tang Y. Hojatkashani C. Dinov I. D. Sun B. Fan L. Lin X.. (2010). The construction of a Chinese MRI brain atlas: A morphometric comparison study between Chinese and Caucasian cohorts. Neuroimage 51, 33–41. 10.1016/j.neuroimage.2010.01.111 20152910 PMC2862912

[B41] Taylor P. Etzel J. A. Glen D. Reynolds R. C. Moraczewski D. Basavaraj A. (2023). FMRI Open QC Project. 10.17605/OSF.IO/QAESM PMC10264898 37325035

[B42] Wei D. Zhuang K. Ai L. Chen Q. Yang W. Liu W.. (2018). Structural and functional brain scans from the cross-sectional Southwest University adult lifespan dataset. Sci. Data 5, 180134. 10.1038/sdata.2018.134 30015807 PMC6049036

[B43] Whitfield-Gabrieli S. Nieto-Castanon A. (2012). Conn: A functional connectivity toolbox for correlated and anticorrelated brain networks. Brain Connect. 2, 125–141. 10.1089/brain.2012.0073 22642651

[B44] Williams B. Lindner M. (2020). pyfMRIqc: A software package for raw fMRI data quality assurance. J. Open Res. Softw. 8, 23. 10.5334/jors.280

[B45] Winkler A. M. Ridgway G. R. Webster M. A. Smith S. M. Nichols T. E. (2014). Permutation inference for the general linear model. Neuroimage 92, 381–397. 10.1016/j.neuroimage.2014.01.060 24530839 PMC4010955

[B46] Xie Y. (2014). “Knitr: A comprehensive tool for reproducible research in R,” in Implementing Reproducible Research, The R Series. eds V. Stodden, F. Leisch, and R. D. Peng (Boca Raton, FL: CRC Press, Taylor & Francis Group).

[B47] Yan C. G. Wang X. D. Lu B. (2021). DPABISurf: Data processing & analysis for brain imaging on surface. Sci. Bullet. 66, 2453–2455. 10.1016/j.scib.2021.09.016 36654202

[B48] Yan C. G. Wang X. D. Zuo X. N. Zang Y. F. (2016). DPABI: Data processing and analysis for (resting-state) brain imaging. Neuroinformatics 14, 339–351. 10.1007/s12021-016-9299-4 27075850

[B49] Yan C. G. Zang Y. F. (2010). DPARSF: A MATLAB toolbox for “pipeline” data analysis of resting-state fMRI. Front. Syst. Neurosci. 14, 4. 10.3389/fnsys.2010.00013 20577591 PMC2889691

[B50] Yoneyama N. Watanabe H. Kawabata K. Bagarinao E. Hara K. Tsuboi T.. (2018). Severe hyposmia and aberrant functional connectivity in cognitively normal Parkinson's disease. PLoS One 13, e0190072. 10.1371/journal.pone.0190072 29304050 PMC5755765

