# Supplementary material for: Editorial: Demonstrating quality control (QC) procedures in fMRI
Source: Front Neurosci. 2023 May 31;17:1205928. doi: 10.3389/fnins.2023.1205928 (PMC10264898; doi:10.3389/fnins.2023.1205928)
Supplement: Supplementary file 1 [file Data_Sheet_1.docx]

**Supplementary Info**

Here we provide the full mapping of names for each subject included in the Research Topic, "Demonstrating quality control (QC) procedures in fMRI." See the main paper for details.

**Group 0 ON, ds000030**

sub-001 sub-10524

sub-002 sub-10530

sub-003 sub-10629

sub-004 sub-10631

sub-005 sub-10678

sub-006 sub-10692

sub-007 sub-10708

sub-008 sub-10958

sub-009 sub-10977

sub-010 sub-11097

sub-011 sub-11143

sub-012 sub-50004

sub-013 sub-50014

sub-014 sub-50021

sub-015 sub-50053

sub-016 sub-50069

sub-017 sub-60014

sub-018 sub-60017

sub-019 sub-60020

sub-020 sub-60042

sub-021 sub-60043

sub-022 sub-60052

sub-023 sub-60053

sub-024 sub-60078

sub-025 sub-60080

sub-026 sub-70020

sub-027 sub-70051

sub-028 sub-70057

sub-029 sub-70072

sub-030 sub-70077

**Group 1 ABIDE-1, KKI**

sub-101 sub-0050775

sub-102 sub-0050776

sub-103 sub-0050777

sub-104 sub-0050781

sub-105 sub-0050782

sub-106 sub-0050787

sub-107 sub-0050788

sub-108 sub-0050790

sub-109 sub-0050802

sub-110 sub-0050809

sub-111 sub-0050812

sub-112 sub-0050813

sub-113 sub-0050814

sub-114 sub-0050816

sub-115 sub-0050817

sub-116 sub-0050819

sub-117 sub-0050820

sub-118 sub-0050821

sub-119 sub-0050822

sub-120 sub-0050824

**Group 2 ABIDE-1, Trinity**

sub-201 sub-0050233

sub-202 sub-0050234

sub-203 sub-0050237

sub-204 sub-0050243

sub-205 sub-0050244

sub-206 sub-0050248

sub-207 sub-0050249

sub-208 sub-0050250

sub-209 sub-0050254

sub-210 sub-0050255

sub-211 sub-0050260

sub-212 sub-0050261

sub-213 sub-0050268

sub-214 sub-0050270

sub-215 sub-0050271

sub-216 sub-0051133

sub-217 sub-0051135

sub-218 sub-0051137

sub-219 sub-0051139

sub-220 sub-0051142

**Group 3 ABIDE-2, KUL_3**

sub-301 sub-29665

sub-302 sub-29666

sub-303 sub-29667

sub-304 sub-29668

sub-305 sub-29669

sub-306 sub-29670

sub-307 sub-29671

sub-308 sub-29678

sub-309 sub-29679

sub-310 sub-29794

sub-311 sub-29795

sub-312 sub-29796

sub-313 sub-29797

sub-314 sub-29798

sub-315 sub-29799

sub-316 sub-29800

**Group 4 FCP, Baltimore**

sub-401 sub-17017

sub-402 sub-19738

sub-403 sub-23750

sub-404 sub-23927

sub-405 sub-29158

sub-406 sub-30072

sub-407 sub-31837

sub-408 sub-37548

sub-409 sub-52358

sub-410 sub-54257

sub-411 sub-54329

sub-412 sub-73823

sub-413 sub-76160

sub-414 sub-77572

sub-415 sub-80221

sub-416 sub-81887

sub-417 sub-85922

sub-418 sub-86414

sub-419 sub-90658

sub-420 sub-90893

sub-421 sub-91622

sub-422 sub-94042

sub-423 sub-96234

**Group 5 ON, ds000220**

sub-501 sub-control01

sub-502 sub-control02

sub-503 sub-control03

sub-504 sub-control06

sub-505 sub-control07

sub-506 sub-control09

sub-507 sub-control10

sub-508 sub-control11

sub-509 sub-tbi01

sub-510 sub-tbi02

sub-511 sub-tbi03

sub-512 sub-tbi05

sub-513 sub-tbi06

sub-514 sub-tbi08

sub-515 sub-tbi09

sub-516 sub-tbi10

sub-517 sub-tbi11

sub-518 sub-tbi12

sub-519 sub-tbi13

sub-520 sub-tbi14

**Group 6 ON, ds000243**

sub-601 sub-001

sub-602 sub-002

sub-603 sub-003

sub-604 sub-006

sub-605 sub-007

sub-606 sub-009

sub-607 sub-011

sub-608 sub-016

sub-609 sub-017

sub-610 sub-020

sub-611 sub-026

sub-612 sub-028

sub-613 sub-038

sub-614 sub-043

sub-615 sub-044

sub-616 sub-045

sub-617 sub-047

sub-618 sub-048

sub-619 sub-064

sub-620 sub-071

**Group 7 ON, ds000245**

sub-701 sub-CTL04

sub-702 sub-CTL08

sub-703 sub-CTL09

sub-704 sub-CTL10

sub-705 sub-CTL14

sub-706 sub-CTL15

sub-707 sub-ODN02

sub-708 sub-ODN04

sub-709 sub-ODN05

sub-710 sub-ODN07

sub-711 sub-ODN08

sub-712 sub-ODN11

sub-713 sub-ODN12

sub-714 sub-ODN14

sub-715 sub-ODP02

sub-716 sub-ODP03

sub-717 sub-ODP06

sub-718 sub-ODP09

sub-719 sub-ODP13

sub-720 sub-ODP14
